# Trauma-focused EMDR for Personality disorders among Outpatients (TEMPO): study protocol for a multi-centre, single-blind, randomized controlled trial

**DOI:** 10.1186/s13063-022-06082-6

**Published:** 2022-03-04

**Authors:** Simon Hofman, Laurian Hafkemeijer, Ad de Jongh, Annemieke Starrenburg, Karin Slotema

**Affiliations:** 1 Department of Personality Disorders, Parnassia Psychiatric Institute, Lijnbaan 4, 2512VA, The Hague, The Netherlands; 2 Department of Adult Psychiatry, GGZ Delfland, Delft, The Netherlands; 3 Academic Centre for Dentistry Amsterdam (ACTA), University of Amsterdam and VU University Amsterdam, Amsterdam, The Netherlands; 4 Research Department, PSYTREC, Bilthoven, The Netherlands; 5 School of Health Sciences, Salford University, Manchester, UK; 6 Institute of Health and Society, University of Worcester, Worcester, UK; 7 School of Psychology, Queen’s University Belfast, Belfast, Northern Ireland; 8 Department of Psychology, Education and Child Studies, Erasmus University Rotterdam, Rotterdam, The Netherlands

**Keywords:** EMDR, Personality disorder, Trauma, Effectiveness, Cost-effectiveness, Economic evaluation, Predictors, Treatment experiences, Study protocol, Randomized controlled trial

## Abstract

**Background:**

Existing recommended treatment options for personality disorders (PDs) are extensive and costly. There is emerging evidence indicating that trauma-focused treatment using eye movement desensitization and reprocessing (EMDR) therapy aimed at resolving memories of individuals’ adverse events can be beneficial for this target group within a relatively short time frame. The primary purpose of the present study is to determine the effectiveness of EMDR therapy versus waiting list in reducing PD symptom severity. Furthermore, the effects of EMDR therapy on trauma symptom severity, loss of diagnosis, personal functioning, quality of life, and mental health outcomes will be determined. In addition, the cost-effectiveness of EMDR therapy in the treatment of PDs is investigated. Moreover, predictors of treatment success, symptom deterioration and treatment discontinuation will be assessed. Lastly, experiences with EMDR therapy will be explored.

**Method:**

In total, 159 patients with a PD will be included in a large multicentre single-blind randomized controlled trial. The Structured Clinical Interview for DSM-5 Personality Disorders will be used to determine the presence of a PD. Participants will be allocated to either a treatment condition with EMDR therapy (ten biweekly 90-min sessions) or a waiting list. Three months after potential treatment with EMDR therapy, patients can receive treatment as usual for their PD. All participants are subject to single-blinded baseline, post-intervention and 3-, 6- and 12-month follow-up assessments. The primary outcome measures are the Assessment of DSM-IV Personality Disorders and the Clinician-Administered PTSD Scale for DSM-5. For cost-effectiveness, the Treatment Inventory of Costs in Patients with psychiatric disorders, EuroQol-5D-3L, and the Mental Health Quality of Life Questionnaire will be administered. The PTSD Checklist for DSM-5, Brief State Paranoia Checklist and Difficulties in Emotion Regulation Scale will be used to further index trauma symptom severity. Type of trauma is identified at baseline with the Childhood Trauma Questionnaire-SF and Life Events Checklist for the DSM-5. Personal functioning and health outcome are assessed with the Level of Personality Functioning Scale-BF 2.0, Outcome Questionnaire-45 and Mental Health Quality of Life Questionnaire. Experiences with EMDR therapy of patients in the EMDR therapy condition are explored with a semi-structured interview at post-intervention.

**Discussion:**

It is expected that the results of this study will contribute to knowledge about the effectiveness, and cost-effectiveness of trauma-focused treatment using EMDR therapy in individuals diagnosed with a PD. Follow-up data provide documentation of long-term effects of EMDR therapy on various outcome variables, most importantly the reduction of PD symptom severity and loss of diagnoses.

**Trial registration:**

Netherlands Trial Register NL9078. Registered on 31 November 2020

## Administrative information

**Table:** 

Title	Trauma-focused EMDR for Personality disorders among Outpatients (TEMPO): Study Protocol for a Randomized Controlled Trial
Trial registration	Netherlands Trial Register: NL9078. Date of approval: 31-11-2020
Protocol version	2, 29-11-2021
Funding	Internal funding by Parnassia Groep. Independent study grant by Vereniging EMDR Nederland. Independent study grant by EMDR Europe.
Author details	1. Simon Hofman: Department of Personality Disorders, Parnassia Psychiatric Institute, The Hague, The Netherlands 2. Laurian Hafkemeijer: Department of Adult Psychiatry, GGZ Delfland, Delft, The Netherlands 3. Ad de Jongh: Academic Centre for Dentistry Amsterdam (ACTA), University of Amsterdam and VU University Amsterdam, Amsterdam, The Netherlands; Research Department, PSYTREC, Bilthoven, The Netherlands; School of Health Sciences, Salford University, Manchester, UK; Institute of Health and Society, University of Worcester, Worcester, UK; School of Psychology, Queen’s University Belfast, Belfast, Northern Ireland. 4. Annemieke Starrenburg: Department of Adult Psychiatry, GGZ Delfland, Delft, The Netherlands 5. Karin Slotema: Department of Personality Disorders, Parnassia Psychiatric Institute, The Hague, The Netherlands; Department of Psychology, Education and Child Studies, Erasmus University Rotterdam, the Netherlands • Corresponding author: C.W. Slotema, Department of Personality Disorders, Parnassia Psychiatric Institute, Lijnbaan 4, 2512VA, The Hague, The Netherlands, e-mail: c.slotema@psyq.nl, phone: +31 88 3573107.
Name and contact information for the trial sponsor	Parnassia Psychiatric Institute, Bestuurscentrum, Monsterseweg 93, 2553RJ, The Hague, The Netherlands, e-mail: RvB-sec@parnassiagroep.nl, phone: +31 88 357 00 10. GGZ Delfland, Raad van Bestuur, Sint Jorisweg 2, 2612GA, Delft, The Netherlands, e-mail: info@ggz-delfland.nl, phone: +31 15 260 76 07.
Role of sponsor	The Parnassia Psychiatric Institute and GGZ Delfland have no authority regarding any of the activities related to the current study.
Committees	Data will be processed in a data management system (Research manager), which facilitates monitoring. Via this system an Audit-trial will be created and a monitor will be assigned. Independently from the researchers and sponsor, this person will monitor the study via the research manager data management system. Furthermore, the monitor will visit the site with a minimum frequency of one visit per year. After the study, a close-out visit will be planned. The monitor will check whether study procedures are followed correctly, and will check the study site's documentation, the participants' source data, electronic Case Report Form (eCRF) entries, and the correct maintenance of the Investigator Site File. At the site, monthly research meetings take place. In these meetings, the project will be discussed with the steering committee. Furthermore, the sponsor will submit progress and safety reports to the Medical Research Ethics Committee (MREC) once a year, informing them of the numbers of inclusions and completions, adverse reactions and other important information concerning the safety of patients. According to the guidelines on data monitoring committees [26], a Data Monitoring Committee (DMC) might not be implemented in studies with non-critical indications or when the intervention under investigation is characterized and known for not harming patients. EMDR therapy is an evidence-based treatment for PTSD and has been found to be safe in patients with a personality disorder. A (DMC) is therefore not instituted.

## Introduction

Patients diagnosed with a personality disorder (PD) often report that they were exposed to adverse childhood events [7]. Most studies in this area include patients with borderline personality disorder (BPD) of which many experienced verbal (72%), physical (46%) and sexual (26%) abuse [72]. A recent study showed that 97% of BPD patients reported at least one type of childhood trauma, including abuse and neglect [56]. In other types of PDs, rates of childhood maltreatment are high as well, with 73% reporting abuse and 82% neglect [7]. Therefore, it is no surprise that prevalence rates of posttraumatic stress disorder (PTSD) among PDs are high, ranging from 41 to 56% in BPD patients [56, 71] and from 20 to 47% in patients with other PDs [29, 70, 71]. Also, prospective research has established associations between childhood maltreatment and development of PDs. For example, data from the longitudinal Children in the Community Study showed that adverse childhood experiences significantly predicted the development of a PD in later life (OR = 6.83) [15, 36, 39, 40]. Taken together, these results point to the importance of adverse childhood experiences and their influence on the development and severity of PD symptoms later in life.

Due to impairments in cognition, emotion regulation and impulse control [44], PD patients experience reduced interpersonal functioning, quality of life and subjective well-being [18]. In a large sample of PD patients, severe impairments in quality of life were found, comparable to the burden associated with rheumatic diseases, lung cancer and Parkinson’s disease [60]. Furthermore, longitudinal evidence suggests that adolescent PDs predict dysfunctional outcomes (e.g. social impairment, suicide, violence) later in life [17]. More specifically, cluster A symptoms were found to be significantly related to a delayed formation of residential, career, financial, romantic and family roles [16, 17], cluster B symptoms to adult aggressive, criminal, impulsive and self-destructive behaviour [38, 17], while cluster C symptoms predicted suicide attempts and partner conflict in early adulthood [14, 37, 17]. Besides these adverse effects stemming from the presence of PDs, a large proportion of individuals with PD are diagnosed with comorbid mental health conditions (67––97% [44]) that exert additive negative effects on functional outcome measures [19]. It is therefore understandable that societal costs of PDs are high (e.g. estimated €11,126 over 1 year prior to treatment [59]). Particularly the recommended treatment options for BPD proved to be extensive and costly [51]. To illustrate, in eight systematically reviewed randomized controlled trials (RCTs) [51], the mean duration of various PD treatments ranged from 168 [41] to 504 days (Bateman, [6]), with an average of 358 treatment days per patient. Moreover, such extensive treatment tracks require (partial) hospitalization or multiple appointments per week [51], further increasing intervention costs.

It has been argued that societal costs of PDs could be greatly reduced by treating this patient group [25]. For example, in 24 patients admitted for treatment at a mental health hospital, a reduction per patient was found involving in-patient, out-patient and prison costs, from £13,966 over 1 year prior to treatment to £1,308 over 1 year following discharge from treatment [25]. Although it is not clear from the description of this study how long and costly these treatments were, these results suggest that if there are good, targeted treatments that can alleviate the symptoms of PD, this would not only curb personal suffering but also reduce and prevent societal costs.

In this regard, there is hope on the horizon. Evidence suggests that (trauma-focused) psychotherapies of short duration might be effective in the treatment of PDs. For example, a recent meta-analysis demonstrated a strong decline in BPD symptoms (Hedges’ *g* = .52), when PTSD patients with comorbid BPD were treated with 4 to 25 sessions of trauma-focused therapies lasting 50 to 120 min [58]. Two studies used treatment with eye movement desensitization and reprocessing (EMDR), a therapy of first choice for PTSD [9, 32, 69]. In treating PTSD, EMDR therapy has been found to be well tolerated and cost-effective [21, 24, 48, 49]. Furthermore, an uncontrolled study showed that a median of four sessions of EMDR therapy was beneficial and safe in patients with comorbid PD and PTSD, where no difference in efficacy of EMDR therapy was found between patients with BPD and other PDs [57]. The effectiveness of EMDR therapy on PD symptom severity has also been studied in samples of patients with PDs but without a PTSD diagnosis. For instance, the results of a RCT comparing five sessions of EMDR therapy to a waiting list in patients diagnosed with any PD, but without PTSD, revealed medium to large effect sizes for psychological symptoms (Cohen’s *d* = .65), psychological functioning (Cohen’s *d* = .62) and personality functioning (Cohen’s *d* = .56), without the occurrence of adverse events [33]. These findings are particularly important because the length of treatment was brief in that patients received a mere total of 7.5 h of EMDR therapy. Yet, gains were maintained at three-month follow-up [33]. The findings of a recent study support the notion that treatment duration of PDs, like BPD, can even be shorter. It was found that a significant reduction of BPD symptoms was established, using a combination of EMDR therapy and prolonged exposure. Interestingly, one third of the patients lost their positive screen for BPD post-treatment after only eight treatment days [22]. These results suggest that EMDR therapy can be effectively, safely and more affordably applied to treat symptoms of PDs. However, until now, studies regarding the effects of EMDR therapy for the symptoms of PDs are limited to open label trials. Accordingly, the next step is to perform an RCT comparing the effectiveness of EMDR therapy versus no treatment intended to control for spontaneous recovery.

Even if we are able to determine that a large proportion of patients with PD can be helped with good treatment, it is still important, for improvement of treatment and cost-effectiveness, to understand which factors predict treatment success and discontinuation. To this end, several predictors have already been identified. Namely, a systematic review showed that, in patients with a PD, higher pre-treatment symptom severity and patient-rated therapeutic alliance significantly predicted greater symptom reduction [5]. Another review found that 37% of 2516 PD patients discontinued treatment which was associated with a series of patient characteristics including younger age, lower education, lower occupational levels, amount of PD diagnoses/criteria, worse general functioning and substance use [50]. In addition, a recent study found that patients with more severe childhood physical abuse were significantly more likely to discontinue treatment [3]. Unfortunately, one of the major problems with this extensive list of predictors is the lack of replication [3]. Additionally, predictive factors of therapy success and discontinuation of EMDR therapy for patients with a PD have not yet been investigated. Thus, in short, while these findings are indicative, they are certainly not conclusive.

To fill these gaps in the literature, the present study, the Trauma-focused EMDR for Personality disorders among Outpatients (TEMPO) study, will be conducted. The purpose of this study is to determine the effectiveness of EMDR therapy in comparison to a waiting list control condition in reducing PD symptom severity in a sample of patients diagnosed with a PD. Furthermore, the effects of EMDR therapy on trauma symptom severity, loss of diagnosis, personal functioning, quality of life and mental health outcomes will be determined. In addition, the cost-effectiveness of EMDR therapy in the treatment of PDs is investigated. Moreover, predictors of treatment success and dropouts will be assessed. Lastly, experiences with EMDR therapy will be explored.

## Method

### Design

**Fig. 1:**
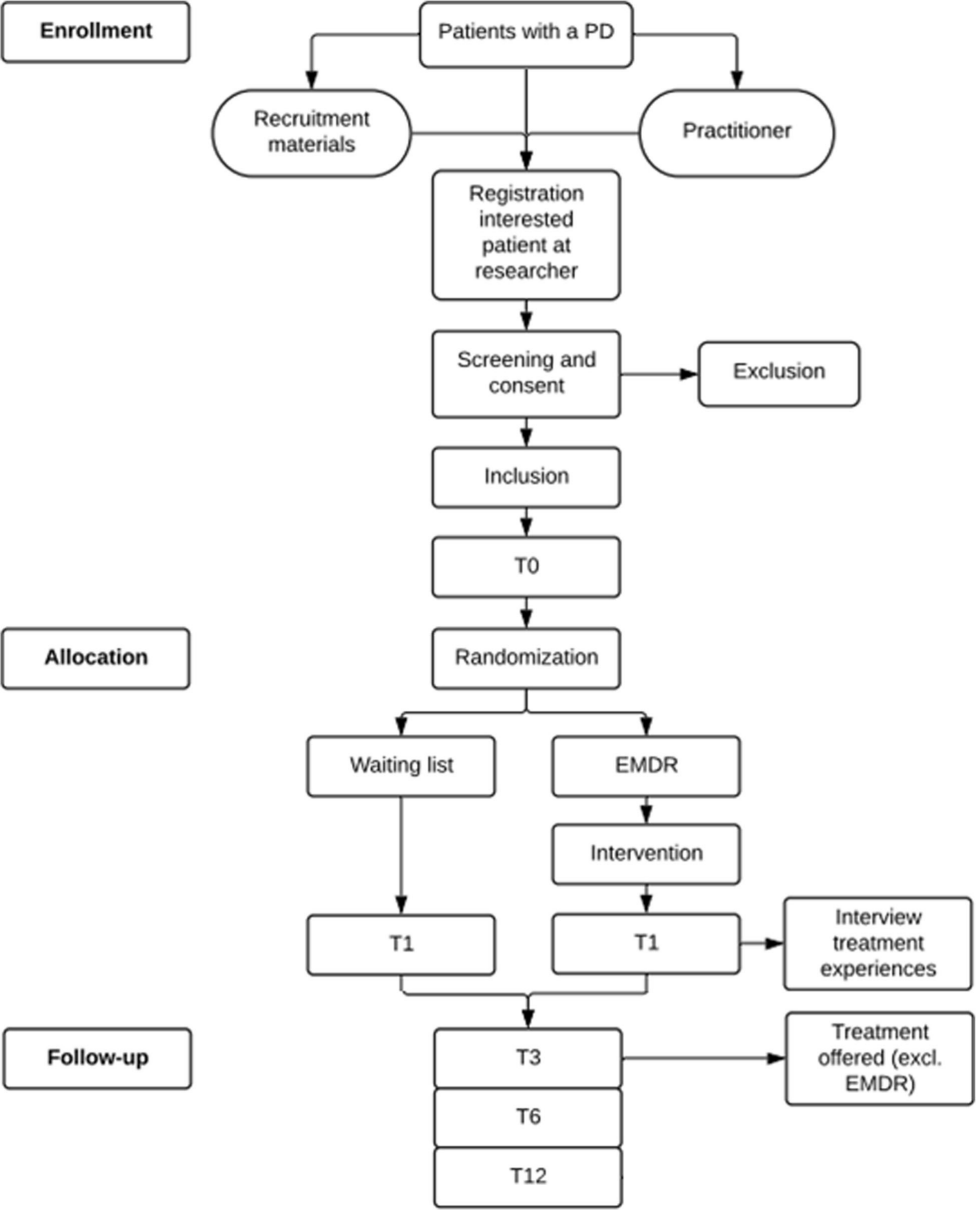
Participant trajectory

The TEMPO study is a single-blind randomized controlled superiority trial with two arms, i.e. EMDR therapy and waiting list. The trajectory of a participant through the study is shown in Fig. 1. The experimental group will receive ten bi-weekly EMDR sessions of 90 min each, with a total duration of 15 h of treatment over 5 weeks. The groups are compared at baseline (pre-randomization; T0), post-intervention (after 5 weeks; T1), 3-month (T3), 6-month (T6) and 12-month (T12) follow-up. At baseline, demographical data will be collected for predictive analyses of treatment success and treatment dropout. At T1, patient dropout will be recorded and a random selection of patients in the experimental condition will be interviewed to assess their experiences with EMDR therapy. Until T3, patients do not receive further treatment, except in a crisis situation. At T3, all patients will be invited by their therapist for a consultation to examine whether (further) treatment is necessary. Any available treatment, except EMDR therapy, will be offered. At T12, when participation is concluded, control patients will also be offered the opportunity to receive EMDR therapy.

For all measurement points, the assessing research assistants are blind to the participant’s research condition. The design of this study was approved by the Medical Research Ethics Committee (MREC) of the Erasmus Medical Centre in Rotterdam, number MEC-2020-0583.

### Participants

All participants (*N* = 159) will be recruited from outpatient clinics of the Parnassia Psychiatric Institute and GGZ Delfland, two institutions for mental health care in the Netherlands. To be eligible to participate in this study, a subject must be (1) at least eighteen years of age and (2) classified with any PD with the aid of the Structured Clinical Interview for DSM-5 Personality Disorders (SCID-5-P). To ensure that patients can competently decide to participate and answer the questions during the clinical interviews or the self-report questionnaires, patients will be excluded if their competency in the Dutch language is inadequate or if their IQ is estimated as lower than 70 (mental disability). The aim is to include a broad range of patients with PDs, because this represents the population that is treated within outpatient specialized mental health care centres for PDs the best.

### Recruitment

Participants who are not yet in treatment will be recruited in three ways. Firstly, in the waiting rooms of the mental health institutions that participate in this study, posters will be placed that briefly explain the goal, design and trajectory of the study. Patients can announce themselves to their therapist or contact the researchers directly. Secondly, patients with a PD will also be informed about the study by their therapist. Thirdly, researchers can directly contact patients who have given approval to being approached for research. If the patient is interested, the therapist informs the researchers, who will send them information via e-mail and invite them for an appointment with one of the researchers. Patients will receive further information about the study during the appointment and will be asked to sign an informed consent form. If consent has been given, the researcher will check eligibility for inclusion by conducting the SCID-5-P. Next, another appointment will be scheduled for baseline measurements.

Participants will receive a financial incentive of 25 euros per complete measurement, potentially totalling 125 euros for all five measurements. They receive this fee at the end of participation (i.e. completion or termination).

### Randomization

Prior to study initiation, a randomization table with 159 rows (participants) and one column (condition: 0 vs. 1; EMDR vs. waiting list; 1:1 ratio without blocks) is generated in Data Manager, an external service for administering questionnaires and safely storing responses, available at Parnassia Psychiatric Institute. Thusly, the condition of any participant is determined beforehand. After a participant has completed the baseline measurements, a case report form (CRF) is manually generated in Data Manager. The non-blind researcher can reveal the participant’s condition by pressing a randomize-button. Participants will be informed of their allocation through telephone or video calls. In Data Manager, it is pre-programmed that conditions are visible for only the non-blind researchers. In addition, the principal investigator (KS) has insight into conditions of all participants. Therefore, unblinding will never be necessary.

### Sample size calculation

In order to test the effectiveness of EMDR in reducing the number of PD symptoms over time, relative to waiting list, a linear mixed model (LMM) with intention-to-treat will be used. A RCT of five EMDR sessions revealed an effect size of .65 for change in psychological symptoms, .62 for psychological functioning and .56 for change in personality functioning [33]. Since change in personality functioning is also (primarily) assessed in the current study, although with the aid of a different measure (Assessment of DSM IV Personality disorders; ADP-IV), the effect size for the main analysis is expected to approximate the effect size found by Hafkemeijer et al. [33]. With the aid of standardization by post score standard deviations (standard procedure for meta-analyses), this effect size decreases to .46. Therefore, an effect size of .43 was estimated to be appropriate for this study. Using Liu and Liang’s [45] equation, assuming a correlation of .7 between five repeated measurements, a treatment effect size of .43, a power of .8, an *α* significance level of .05, and two treatment conditions, the power analysis resulted in a total sample size of 132 patients. With an estimated dropout rate of 20%, a total of 159 patients is needed.

### Conditions

#### EMDR therapy

Half of the participants will receive EMDR therapy. This is a standardized, eight-phase, trauma-focused therapy. The EMDR standard protocol incorporates several predetermined steps, consisting of standard questions and formulations intended as process instructions. Further, EMDR therapy consists of dosed attention directed at the disturbing memory, at the same time engaging in another concurrent (dual-attention) task [24, 55]. The task typically requires patients to follow the horizontally moving fingers of the therapist with their eyes, but taps, tones and other tasks that tax patients’ working memory [4] can also be applied. For a full description of the treatment protocol, see Shapiro [55].

In the current study, a total of ten biweekly sessions of EMDR therapy will be applied with a duration of 90 min per session. Before onset, a case conceptualization is made to guide the treatment sessions. If patients report having been exposed to traumatic events (i.e. criterion A of PTSD [1]), the EMDR sessions will target the associated traum atic memories first (intrusive before non-intrusive). If there is no clear trauma, the EMDR therapy sessions will target the memories that gave rise to or worsened the patient’s most distressing symptoms. All memories will be placed in a hierarchy based the subjective units of disturbance (SUD) and will be treated from high to low SUD. Each case conceptualization will be review ed by a highly experienced and qualified supervisor prior to the beginning of therapy. During the sessions, the Dutch version of the standard EMDR protocol [23] will be used. Participants are asked to focus on the currently most distressing memory, includin g images, thoughts, emotions and physical sensations. Besides eye movements, other forms of working memory taxation, such as hand taps or tones, are used to process the memories. 

Protocol adherence and treatment quality are improved in a number of ways. First, EMDR therapy will be provided by psychologists, psychiatrists and residents in psychiatry who have attended at least the official Dutch Basic EMDR training and in this way are certified according to the guidelines of the EMDR Europe association (EMDREA). Secondly, in advance of study initiation, all therapists need to attend an additional one-day training aimed at case conceptualization and to improve their skills needed to treat patient s suffering from a PD, provided by an EMDREA accredited trainer (AdJ). Thirdly, monthly group consultations will be organized by EMDREA approved consultants. Fourthly, these consultants review the EMDR sessions through session reports that the therapists share with the consultants directly following each session. Additional consultation is available on request. Fifthly, all treatment sessions will be videotaped and coded. Therapists are obligated to show parts of their recordings during the monthly consultation sessions, so that therapists’ skills can be further improved. Lastly, 10% of all recordings will be selected (using www.randomization.com) and rated for therapist competence and treatment protocol adherence by trained and blinded assessors (EMDREA approved consultants) after conclusion of the trial.

A participant will be considered an early completer of EMDR therapy when the subjective levels of disturbance associated with the memories that were part of the case conceptualization have decreased to zero before the ten sessions have been completed, and the participant no longer meets the diagnostic criteria for a PD according to the ADP-IV. At this point, therapy is terminated. However, post-intervention data will still be collected at approximately the same moment as non-early completers. It is expected that a large proportion of the participants are able to process multiple memories per session during the ten protocolled therapy sessions. This may lead to some of the patients being considered “early completers”. In some cases, it may be decided to (temporarily) discontinue therapy, for which the following criteria apply: (1) participant request, (2) decision by primary caretaker, (3) decision by the supervising EMDR consultants or (4) admission to crisis mental health care.

After 3-month follow-up (T3), the therapist who did the diagnostic procedure on admission and the participant will confer about the current state of the participant, whether further treatment as usual for the PD is necessary and, if so, which treatment is most suitable. These treatments might be emotion regulation training and psychotherapy for PD, including schema- or dialectical behaviour therapy. However, any PD treatment is postponed until after T3.

#### Waiting list

The other half of the participants will be allocated to the control condition, namely a waiting list. They will not receive EMDR therapy and any other treatment for their PD is postponed until after T3 as well. They will be informed of their condition right away and invited for another appointment for post-intervention measurements (T1). Three months later, besides T3 assessments, all participants will confer with the therapist who did the diagnostic procedure on admission, to decide which treatment is most suitable. Patients will be offered the opportunity of following any available treatment, but not EMDR therapy, for their PD. The patient will be registered for the chosen treatment based on the date of intake so that the patient does not have additional waiting time. At study termination (T12), participants in the control condition will also be offered EMDR therapy.

### Adverse events

All adverse events reported spontaneously by the subject or observed by the investigator or his staff will be recorded. The investigator will report all serious adverse events (SAEs) to the sponsor without undue delay after obtaining knowledge of the events. The sponsor will report the SAEs through the web portal *ToetsingOnline* to the accredited medical ethical review board that approved the protocol, within 7 days of first knowledge for SAEs that result in death or are life threatening followed by a period with a maximum of 8 days to complete the initial preliminary report. All other SAEs will be reported within a period of maximum 15 days after the sponsor has first knowledge of the serious adverse events.

### Measurements

There will be five measurement moments, namely at baseline (T0), post-intervention (T1), 3-month follow-up (T3), 6-month follow-up (T6) and 1-year follow-up (T12). While most measures will be conducted at each measurement moment (see Table 1), the Childhood Trauma Questionnaire and the Life Events Checklist will only be administered at baseline and the interview about patients’ experiences with EMDR therapy is exclusively conducted at T1, with only the experimental group. All measurements will take place at the location at which the participant is in treatment or through video calls. The self-report questionnaires will be digitally administered with Data Manager, which automatically invites participants through email on pre-determined dates and then stores the responses safely. Participants will be reminded to fill in the questionnaires, first by mail and second by telephone, to promote complete follow-up data. At baseline, demographical data are collected. Results will be recorded as absolute values.

**Table 1:** Schedule of enrolment, interventions and assessments

Timepoint	Study period							
				Post-allocation				
	Enrolment	T0	Allocation	Intervention	T1	T3	T6	T12
**Enrolment:**								
***Eligibility screen***	x							
***Informed consent***	x							
***Allocation***			x					
**Interventions:**								
***EMDR***				x				
***Waiting list***								
**Assessments:**								
***ADP-IV***		x			x	x	x	x
***CAPS-5***^**a**^		x			x	x	x	x
***SCID-5-P***^**a**^	x				x	x	x	x
***TiC-P***		x			x	x	x	x
***EQ-5D-3L***		x			x	x	x	x
***MHQoL***		x			x	x	x	x
***LEC-5***		x						
***PCL-5***		x			x	x	x	x
***CTQ-SF***		x						
***DERS***		x			x	x	x	x
***BSPC***		x			x	x	x	x
***LPFS-BF 2.0***		x			x	x	x	x
***OQ-45***		x			x	x	x	x
***Appendix A/B***^**a**^					x^b^			

*EMDR* Eye Movement Desensitization and Reprocessing, *ADP-IV* Assessment of DSM-4 Personality Disorders, *CAPS-5* Clinician-Administered PTSD scale for DSM-5, *SCID-5-P* Structured Clinical Interview for DSM-5 Personality Disorders, *TiC-P* Treatment Inventory of Costs in Patients with psychiatric disorders, *EQ-5D-3L* EuroQol—5 dimensions—3 levels, *MHQoL* Mental Health Quality of Life, *LEC-5* Life Events Checklist for the DSM-5, *PCL-5* PTSD Checklist for DSM-5, *CTQ-SF* Childhood Trauma Questionnaire—Short form, *DERS* Difficulties in Emotion Regulation Scale, *BSPC* Brief State Paranoia Checklist, *LPFS-BF 2.0* Level of Personality Functioning Scale—Brief Form 2.0, *OQ-45* Outcome Questionnaire-45

^a^Interview

^b^Experimental condition only

#### Primary outcome measure

##### Assessment of DSM-IV Personality Disorders (ADP-IV)

The ADP-IV is a Dutch self-report questionnaire intended for the assessment of PD characteristics [54]. This measure consists of 94 items, with two questions each. First, on a 7-point Likert scale, the participant indicates the presence of a trait (i.e. the trait score). Then, if the trait is present, i.e. rated as five or higher, the participant specifies the associated degree of distress on a 3-point Likert scale (i.e. the distress score). The ADP-IV allows for a categorical diagnosis. A trait is rated as present when the trait score is rated higher than four and the distress score higher than one. The ADP-IV also allows for a dimensional total score by summing all trait scores. The total score has demonstrated good internal consistency (*α* = .85) and the subscales for the specific PDs range from questionable for schizoid PD (*α* = .6) to good for avoidant PD (*α* = .84), with a median acceptable internal consistency (*α* = .77 [54]). Validated translations are available as well.

##### Clinician-Administered PTSD Scale for DSM-5 (CAPS-5)

The CAPS-5 is a structured diagnostic interview used for measuring the severity of PTSD [11]. The CAPS-5 provides severity ratings for twenty DSM-5 PTSD symptoms on a scale of 0 to 5. Summing these severity scores results in a total PTSD severity score. Ten other questions regard the duration of the symptoms, impairment resulting from the symptoms, and dissociative symptoms. The assessors in the current study will be or have already been trained in the administration of the CAPS-5. Psychometric evaluation demonstrates a high internal consistency (*α* = .9) and interrater reliability (ICC = .98 [12]).

#### Secondary outcome measures

##### Structured Clinical Interview for DSM-5 Personality Disorders (SCID-5-P)

The SCID-5-P is a structured diagnostic interview used for the evaluation of DSM-5 PDs [28]. It provides severity ratings of each PD symptom on a 3-point scale. Based on these ratings, a symptom can be assessed as present or absent [2]. In total, 135 questions inquire into the presence, examples and explanations of a symptom. Sometimes, multiple questions refer to the same symptom to capture the diverse aspects of the criterium. The SCID-5-P should exclusively be administered by trained clinicians as it requires clinical interviewing skills and the ability to add questions. The assessors in the current study will be or have already been trained in the administration of the SCID-5-P. Reliability and validity data of the Dutch SCID-5-P are not yet available, but they are expected to equal the nearly identical previous version, the Structured Clinical Interview for DSM-IV Axis II Personality Disorders (SCID-II [68]). Interrater agreement on the relevant scales of the SCID-II appears substantial to almost perfect (*κ* = .78-.98 [46, 47]).

##### Treatment Inventory of Costs in Patients with psychiatric disorders (TiC-P)

The TiC-P is a Dutch self-report questionnaire used for the evaluation of costs related to psychosocial problems, such as a psychologist or medication (http://www.imta.nl/questionnaires/). The TiC-P is divided into three parts. The first part consists of fifteen questions pertaining whether and to which degree the respondent has used certain mental health care utilities in the last 3 months. The second part consists of a shortened version of the Health and Labour Questionnaire. Eleven questions record the degree to which psychosocial problems have hindered the respondent in (paid and unpaid) work. A third part consists of some general questions about the respondent. To reach a total cost score, the frequency of healthcare consumption/productivity loss is multiplied by a monetary value. Test-retest analyses have shown that the TiC-P is reliable, with agreement of the first part ranging from moderate to almost perfect (*κ* = .49–.84) and agreement of the second part being substantial (*κ* = .65–.76 [13];).

##### EuroQol—5 dimensions—3 levels (EQ-5D-3L)

The EQ-5D-3L [27] is a self-report questionnaire that measures generic health. It consists of two parts. The first part assesses health in five dimensions (mobility, self-care), each of which has three levels of responses (no, some or extreme problems). Results can be reported as a health state. For example, someone with health state 11223 experiences no problems in the first and second, moderate problems in the third and fourth and severe problems in the fifth dimension. Health states can then be assigned an index score based on societal preference weights for the health state [43, 65]. The second part consists of a visual analogue scale ranging from zero to hundred or from worst to best imaginable health. Respondents are asked to indicate their health condition. A Dutch translation will be used.

##### Mental Health Quality of life (MHQoL) questionnaire

The MHQoL is a self-report quality of life measure, recently developed for use in people with mental health problems (https://www.imta.nl/mhqol/). It consists of eight items. The first seven items refer to specific aspects of mental health (e.g. independence, relationships). Respondents are asked to rate their degree of satisfaction within these mental health aspects on a four-point Likert scale. By summing the responses on these seven items, an index score is calculated. The eighth item inquiries about general psychical wellbeing with a visual analogue scale, ranging from zero to ten or worst to best imaginable psychical wellbeing. Internal consistency is high for the total score of the Dutch version (*α* = .85 [63]).

##### Life Events Checklist for the DSM-5 (LEC-5)

The LEC-5 is a self-report questionnaire that evaluates potential experiences with a wide array of traumatic events [31]. It consists of seventeen items, each a potentially traumatic event (e.g. assault with weapon). Additionally, it uniquely inquires about various types of exposure to each potentially traumatic event. Concretely, respondents rate their experiences with each event on a five-point Likert scale: 1 = happened to me; 2 = witnessed it; 3 learned about it; 4 = not sure; and 5 = does not apply. Thus, it captures elements that may be overlooked by other measures; learning about a natural disaster, for example, might be quite traumatic. A Dutch version will be used, which has not yet been evaluated. However, agreement for the original scale is substantial (*κ* = .61 [31]).

##### PTSD Checklist for DSM-5 (PCL-5)

The PCL-5 is a self-report questionnaire used for assessing PTSD [66]. On a five-point Likert scale, respondents are asked to indicate to which degree they suffered from twenty PTSD-related problems (e.g. reoccurring unpleasant dreams) in the last month. The problems are divided into four subscales: (1) intrusive recollections, (2) avoidance/numbing, (3) cognition/mood and (4) arousal but can be summed to yield a continuous measure of PTSD symptom severity [10]. A Dutch translation will be used, of which the total score has excellent internal consistency (*α* = .93 [64]).

##### Childhood Trauma Questionnaire—Short Form (CTQ-SF)

The CTQ-SF is a self-report questionnaire intended as a screening measure for maltreatment histories in both clinical and non-referred groups [8]. On five-point Likert scales, respondents are asked to indicate the degree of truth of 25 statements about childhood trauma (e.g. ‘I was molested’ or ‘I had to wear dirty clothes’). The CTQ-SF consists of five subscales: physical and emotional neglect and physical, emotional and sexual abuse. A Dutch translation will be used, of which the scales have good to excellent internal consistency (*α* = .89–.95), except for the physical neglect scale, which has questionable internal consistency (*α* = .63 [62]).

##### Difficulties in Emotion Regulation Scale (DERS)

The DERS is a self-report questionnaire, developed for the measurement of clinically relevant difficulties in emotion regulation [30]. On a five-point Likert scale, the respondent indicates the frequency of 36 emotion regulation-statements (e.g. ‘I know how I feel’, ‘when I am upset, I lose control over my behaviour’). The DERS consists of six subscales, namely: lack of emotional clarity, lack of emotional awareness, impulsivity, non-acceptance of emotional responses, limited access to emotion regulation strategies and difficulties engaging in goal-directed behaviour. Whereas the English DERS has demonstrated excellent internal consistency in a BPD sample (*a* = .94 [30]), the subscales of the Dutch version have shown good internal consistencies in an adolescent sample (average *a* = .81 [52]).

##### Brief State Paranoia Checklist (BSPC)

The BSPC is a self-report questionnaire that measures paranoid thinking [53]. On a ten-point Likert scale, the respondent is asked to indicate the current applicability of five statements regarding paranoid ideation, where a higher score indicates greater paranoid thinking. The scale has demonstrated good internal consistency (*α* = .83 [53]).

##### Level of Personality Functioning Scale—Brief Form 2.0 (LPFS-BF 2.0; LFPS)

The LPFS is a self-report questionnaire intended for assessing personality functioning based on the alternative DSM-5 models for PDs [1, 35]. Specifically, it measures interpersonal and self-functioning, as are its two subscales. On a four-point Likert scale, the respondent is asked to indicate the applicability of twelve statements referring to interpersonal or self-functioning. The internal consistency estimates of the Dutch version are high for the total scale (*α* = .82), the self- (*α* = .79) and interpersonal (*α* = .71) functioning scales [67].

##### Outcome Questionnaire (OQ-45)

The OQ-45 is a self-report questionnaire, devised for repeating measurements of client status through the course and at termination of therapy [42]. With 45 broad-ranging health-related statements, where the respondent indicates their applicability on a five-point Likert scale, it measures three domains of functioning; symptom distress, interpersonal relations and social role. A Dutch translation will be used. In clinical samples, it has demonstrated excellent internal consistency for the total score (*α* = .93), but questionable internal consistency for the social role subscale (*α* = .69) [20].

##### Interview

To evaluate patients’ experiences with EMDR therapy, an interview was designed (see Appendix A and B). A non-blind researcher checks every fifth participant (e.g. 5, 10, 15…) to determine whether this person is in the EMDR treatment condition. If so, the person will be invited for the interview after T1. If not, the researcher will check the next participant (e.g. if participant 15 is in the control condition, the researcher checks participant 16), until an EMDR participant is found. Interested participants will be interviewed by the researcher. In qualitative research, sample sizes are difficult to predict. Namely, in general, inclusion is continued until the interviews provide no further information. Yet, as an estimate, about twenty interviews will be sufficient to answer the research question. The interview consists of 13 open questions, which start generic, but gradually become more and more specific, so that most aspects of their experiences with EMDR therapy are covered.

### Analyses

Mixed models with intention-to-treat will be used to explore the efficacy of EMDR in reducing symptoms of PD. These analyses will be used for the majority of other outcome measures. The cost-effectiveness evaluation will be undertaken according to the Dutch guidelines for cost effectiveness studies taken a societal perspective [34]. The severity of PD (ADP-IV) will be used for effectiveness. The TiC-P is used to collect data on the utilization of medical services and productivity loss. Utility scores are estimated using the EQ-5D-5L and MHQoL will measure the effects. A cost-utility analysis and a cost-effectiveness analysis (CEA) are conducted. The cost-utility is calculated as the incremental costs and utilities, which yields a cost per QALY estimate. The cost-effectiveness is expressed in the incremental costs and scores on the ADP-IV.

Multilevel modelling is used to model cost-effectiveness. For assessing the uncertainty, cost-effectiveness acceptability curves and cost-effectiveness planes will be created after bootstrapping. The acceptability curve illustrates the probability that the cost-effectiveness ratio will be accepted for different thresholds. In a cost-effectiveness plane, both incremental costs and incremental effects are plotted to account for combinations. Sensitivity analyses will be performed after the analysis if necessary.

Subgroup analyses will be performed to further investigate the effectiveness of EMDR therapy in patients with or without PTSD or with BPD or any other PD.

Recordings will be made of the qualitative interviews. All transcripts will be summarized by the researchers and provided to participants for respondent validation. The researchers will thoroughly read all transcripts, highlight all potentially relevant excerpts and sort them based on the research questions. These sorted data will then be independently coded by the researchers; excerpts will be grouped based on their similarities. Then, these groupings will be identified/themed. Consequently, the researchers will confer and attempt to reach a consensus on all groupings and themes, resulting in a preliminary codebook. The codebook and two information-rich transcripts as examples will be presented to the study group. Themes will be reviewed, focussing on understanding the data and confirming that the data still correspond to the assigned themes. These themes will form the basis of the conclusions of this sub-study and will be reported.

Predictors of treatment success and discontinuation will be separately assessed with logistic regression analyses and calculated with 5000 bootstraps. Treatment success is defined as a loss of PD diagnosis at T1 in a first analysis, and at T12 in a second analysis. Considering the literature (see introduction [5]) and to test whether effectiveness differs between specific PDs, patients with or without PTSD, the centres of treatment and the EMDR therapists, the following predictors will be entered into a backward logistic regression with a two-tailed significance level of *α* = .05 as criterion: (1) pre-treatment symptom severity, (2) patient-rated therapeutic alliance, (3) specific PS diagnosis, (4) PTSD diagnosis, (5) centre of treatment and (6) EMDR therapist. Considering the literature (see introduction [50, 61]) and to test whether discontinuation differs between specific PDs, patients with or without PTSD, the centres of treatment and the EMDR therapists, the following potential predictors of treatment discontinuation will be entered into a backward logistic regression with a two-tailed significance level of *α* = .05 as criterion: (1) treatment credibility, (2) age, (3) education, (4) occupational levels, (5) amount of PD diagnoses, (6) amount of PD criteria, (7) general functioning, (8) substance use, (9) severity of adverse childhood events, (10) specific PS diagnosis, (11) PTSD diagnosis, (12) centre of treatment and (13) EMDR therapist. Analyses will be checked for robustness by redoing them with survival analysis.

## Discussion

The present study, entitled the Trauma-focused EMDR for Personality disorders among Outpatients (TEMPO) study, is the first single-blind randomized controlled superiority trial that primarily investigates the effectiveness of EMDR therapy on personality disorder (PD) severity in a large sample of PD patients with a follow-up period of 1 year. Based on previous research findings, it is hypothesized that the data of the TEMPO-study will support EMDR therapy as being both an effective and cost-effective treatment for PD. Treatment with EMDR therapy is expected to be associated with a significant reduction in symptoms of PD, a significant decrease in proportion of patients no longer fulfilling the diagnostic criteria of a PD, and an increase in personal functioning, quality of life, and mental health outcomes, evidently favouring the patient. Furthermore, it is hypothesized that the duration of further treatment after EMDR therapy, treatment waiting time and treatment cost will be reduced, favouring the patient group and society at large.

This study has a number of strengths. Firstly, it is a multicentre, outpatient trial that includes a wide variety of PD patients and EMDR therapists, enhancing the external validity of the study. Secondly, a broad range of outcome measures is used in addition to measures of PD symptomatology, such as measures involving cost-effectiveness, and severity of trauma symptoms, which is likely to extend the literature on the effectiveness of EMDR therapy for (sub-threshold) PTSD in comorbid PTSD and PD populations, functioning, quality of life and patients’ experiences with EMDR therapy. Thirdly, the study includes multiple follow-up measurements, documenting the longevity of the hypothesized effects. Fourthly, treatment protocol adherence by EMDR therapists will be evaluated utilising a stopwatch and video recordings. These types of measures can help therapists to stay focused, not to elaborate, and to encourage sticking to the protocol. In addition, it will be possible not only to count the number of sessions, but also to calculate the average therapy time, and of course to determine afterwards to what extent the protocol was applied correctly. Also, some weaknesses of the study should be noted that cannot be avoided for ethical reasons. Firstly, blinding participants in the experimental condition is impossible and participants in the control condition should be informed of their allocation. Expectancy-effects might therefore influence the results. However, at all times, assessing researchers are blinded to the condition the participant is in. Secondly, after three months of follow-up, patients will be offered further treatment. The type of treatment, and therefore the intensity and duration of the treatments, may vary substantially among participants. However, the measurements used for the cost-effectiveness analyses include measures of healthcare consumption, allowing collection of exact data on the interventions that patients receive after T3. With these data, differences in health care utility between the control and experimental group can be explored.

In conclusion, the present study is the first RCT that will determine the effectiveness of EMDR therapy in reducing PD symptom severity and its ability to make patients no longer meet the diagnostics criteria of their PD at 1-year follow-up. If EMDR therapy indeed proves to be (cost-)effective in reducing or abolishing PD symptoms, this would provide a strong argument for including EMDR therapy as a standard treatment option for PDs, thereby significantly reducing treatment duration and costs, evidently benefiting the individual and society at large.

Protocol version 2, 29-11-2021

## Trial status

Inclusion: started 15 February 2021, approximate ending 15 August 2023

## Appendix A: Interview experiences with EMDR

*VOOR DE ONDERZOEKER: We willen inzicht krijgen in de ervaringen van patiënten met EMDR-therapie. De volgende (deels overlappende) vragen kunnen daarbij gesteld worden. De onderzoeker kan vervolgens doorvragen om additionele informatie te vergaren. Het einddoel is om duidelijk te maken hoe de patiënt zijn of haar behandeling met EMDR heeft ervaren.*

1. Hoe kijkt u terug op uw EMDR-behandeling?
2. Vindt u dat uw EMDR-behandeling succesvol is geweest? Waar merkt u dat aan? Welke specifieke klachten zijn verminder of verdwenen?
3. In hoeverre verschilt uw leven van voor en van na de EMDR-behandeling?
4. Had de EMDR-behandeling voor u ook nadelen? Wat was concreet het nadeel?
5. Specifiek, in hoeverre merkte u bijeffecten van de EMDR kort na een sessie?
6. Vond u de voordelen van de behandeling opwegen tegen de nadelen?
7. Heeft de EMDR-behandeling ook een (positief of negatief) effect gehad op uw andere behandelingen? Heeft u ervaringen uit de EMDR-behandeling nader besproken in uw hoofdbehandeling?
8. Wat vond u van het moment waarop u EMDR-behandeling heeft gevolgd? Zou EMDR eerder of later in het proces moeten worden gegeven?
9. Wat vond u van uw EMDR-behandelaar? Wat hielp u en wat niet?
10. Hoe zou u, op een schaal van 1 (verschrikkelijk) tot 10 (uitstekend), de therapeutische band met uw behandelaar beoordelen?
11. Bent u tevreden over de keuze om destijds een EMDR-behandeling te gaan starten? Zou u dezelfde keuze nog eens maken?
12. Zou u het anderen (met dezelfde klachten) aanraden ook een EMDR-behandeling te volgen? Waarom?
13. Heeft u nog verbeteringen voor onze EMDR-behandeling?

## Appendix B: English translation of interview experiences with EMDR

*FOR THE RESEARCHER: We would like to gain insight in patients’ experiences with EMDR therapy. The following (partly overlapping) questions could be used in this regard. The researcher can subsequently dig deeper to retrieve additional information. The end goal is to elucidate how the patient experienced his or her treatment with EMDR.*

1. How do you look back on your treatment with EMDR?
2. Do you think your EMDR treatment was successful? What makes you think that? Which specific complaints have diminished or disappeared?
3. To what degree does your life before differ from your life after EMDR treatment?
4. Were there any cons of your treatment with EMDR? Concretely, what cons did you experience?
5. To what degree did you notice side-effects of EMDR treatment shortly after a session?
6. Did the pros outweigh the cons?
7. If applicable, did the EMDR treatment also have an effect (positive or negative) on other treatments? Did you discuss your experiences with EMDR in your main treatment?
8. What did you think of the moment at which the EMDR treatment was given? Should EMDR be given earlier or later in the process?
9. What did you think of your EMDR therapist? What helped and what did not?
10. How would you, on a scale from 1 (terrible) to 10 (excellent), rate the therapeutic relationship between you and your therapist?
11. Are you satisfied with your choice of starting EMDR treatment? Would you make the same decision again?
12. Would you recommend EMDR therapy to others with the same complaints? Why?
13. Can you think of any improvements for our EMDR therapy?
